# A Scoping Review of CERAMENT™ Applications in Orthopedic Surgery

**DOI:** 10.3390/jcm14217455

**Published:** 2025-10-22

**Authors:** Antonio Bove, Adriano Braile, Sabrina Sirico, Nicola Orabona, Mariantonia Braile

**Affiliations:** 1 Unit of Orthopaedics and Traumatology, Ospedale del Mare, 80147 Naples, Italy; antonio.bove@aslnapoli1centro.it (A.B.); adriano.braile@hotmail.it (A.B.); nicola.orabona@aslnapoli1centro.it (N.O.); 2 Department of Medical and Surgical Specialties and Dentistry, University of Campania “Luigi Vanvitelli”, 81100 Naples, Italy; 3 Department of Clinical Sciences and Translational Medicine, University of Tor Vergata, 00133 Rome, Italy; 4 Medifor, 80013 Naples, Italy; info@mediforsrl.it; 5 Department of Woman, Child and of General and Specialized Surgery, University of Campania “Luigi Vanvitelli”, 81100 Naples, Italy

**Keywords:** antibiotic-loaded bone substitutes, bone void filler, CERAMENT™, osteoconductive materials, orthopedic applications

## Abstract

**Background:** Bone defects resulting from trauma, infection, or benign tumors pose major challenges in orthopedic surgery. Traditional approaches, such as autologous bone grafting, are limited by donor site morbidity and graft availability. CERAMENT™, a synthetic bone substitute composed of calcium sulfate and hydroxyapatite, offers an alternative with osteoconductive properties, controlled resorption, and injectability. **Methods:** A scoping review was conducted in accordance with PRISMA-ScR guidelines. Literature searches were performed in PubMed, Embase, and Scopus through 3 July 2025, using the terms “CERAMENT™” and “Orthopedics.” Studies were selected based on the PICO framework, focusing on clinical applications of CERAMENT™ in human orthopedic procedures. **Results:** Out of 480 initial records, 22 studies met the inclusion criteria. CERAMENT™ demonstrated favorable outcomes in a range of orthopedic settings. In the CERTiFy trial, it was non-inferior to autologous grafting in tibial plateau fractures. CERAMENT™ achieved full wound healing and bone remodeling in chronic osteomyelitis. Additional studies reported positive outcomes in tumor-related defect reconstruction, spinal augmentation, and foot and ankle surgery, highlighting reduced surgical morbidity and faster recovery. **Conclusions:** CERAMENT™ offers a versatile, effective solution for bone reconstruction across multiple orthopedic domains. Its clinical performance, ease of use, and antimicrobial capabilities support its integration into routine orthopedic practice. Further research may refine its indications and long-term benefits.

## 1. Introduction

Bone defects resulting from trauma, infection, or benign bone tumors present significant challenges in orthopedic surgery [1]. Traditional treatment options, such as autologous bone grafting, have limitations including donor site morbidity, limited availability, and potential complications [2]. In response to these challenges, synthetic bone substitutes have been developed to provide effective solutions for bone reconstruction [1,2].

One such innovative material is CERAMENT™ (Bonesupport AB, Lund, Sweden), a synthetic, injectable bone void filler that has garnered attention for its osteoconductive properties and versatility in various orthopedic applications [3,4]. CERAMENT™ is a biodegradable biphasic ceramic composite consisting of hydroxyapatite (HA, 40%) and fast-resorbing calcium sulfate (CaS, 60%) [5], designed to mimic the properties of cancellous bone, enabling controlled resorption that supports vascular ingrowth, stem cell migration, and eventual osteoblast differentiation and bone remodeling [6,7,8]. An HA framework that functions as an osteoconductive scaffold is left behind when the CaS component of the biocomposite normally resorbs over the course of 4 to 6 weeks [8]. By promoting vascular ingrowth, this scaffold makes it possible for stem cells to arrive at the location, develop into osteoblasts, and attach to the HA matrix [8].

To date, CERAMENT™ exists in two antibiotic-loaded varieties that are sold commercially. It is available in 5 mL and 10 mL versions under the names CERAMENT™ G (gentamicin) and CERAMENT™ V (vancomycin) [9]. There are two ways that the resorbable local antibiotic carrier works. In addition to providing a local antibiotic concentration that is higher than the minimum inhibitory concentration (MIC) for the majority of susceptible microorganisms for at least 28 days, it also remodels to bone while maintaining acceptable serum levels [10,11,12]. However, no antibiotic was found in the urine after 30 days, suggesting that all the antibiotics had been eluted by then. The use of PMMA, for example, reduces the chance of low antibiotic concentrations over an extended period of time, which lowers the probability of antibiotic resistance developing [13]. Compared to local injection or intravenous injection, the local administration of the antibiotic-containing ceramics offers a more effective anti-infectious action. The dual functionality of CERAMENT™ G as both a bone substitute and an antimicrobial agent positions it as a valuable tool in the management of infected bone defects [14,15]. CERAMENT™ has been evaluated in various clinical scenarios, demonstrating its efficacy and safety across multiple orthopedic disciplines. In the treatment of tibial plateau fractures, CERAMENT™ has been shown to be non-inferior to autologous bone grafting [16]. The CERTiFy study, a prospective, randomized trial, reported comparable outcomes in terms of bone healing and functional recovery, with the added benefits of reduced surgical morbidity and operative time associated with CERAMENT™ use [3]. In the management of chronic osteomyelitis, CERAMENT™ G has demonstrated significant clinical efficacy [4,14,15,17,18,19,20,21].

Despite the growing body of evidence supporting the use of CERAMENT™ in various orthopedic applications, a comprehensive synthesis of its clinical efficacy and safety across different specialties is lacking. By systematically evaluating the existing literature, this review aims to elucidate the multifaceted applications of CERAMENT™ and its impact on patient outcomes, thereby enhancing its integration into clinical practice and guiding future research endeavors.

## 2. Methods

### 2.1. Information Sources and Search Strategy

A systematic review was conducted to identify all studies reporting outcomes related to the use of CERAMENT™, a synthetic, antibiotic-loaded bone graft substitute composed primarily of CaS and HA, in various orthopedic applications, including bone defect repair and infection management. Literature searches were performed across three major databases: Embase, PubMed, and Scopus. Keywords were combined in various permutations for each database, including terms such as “CERAMENT™” and “Orthopedics”. The database search was completed on 3 July 2025.

To assess the eligibility of each study, the PICO framework was applied as follows: Population (P) consisted of patients receiving CERAMENT™ treatment for orthopedic indications such as osteomyelitis, fracture-related infections, and critical-size bone defects; Intervention (I) was the application of CERAMENT™ as a bone void filler or bone graft substitute; Comparison (C) included other bone graft substitutes or standard surgical treatments; Outcome (O) encompassed clinical efficacy, infection control, bone regeneration, and safety. The scope and methodology of this review adhere to the Preferred Reporting Items for Systematic reviews and Meta-Analyses extension for Scoping Reviews (PRISMA-ScR) [22]. The PRISMA-ScR checklist is reported in Supplementary File S1.

### 2.2. Eligibility Criteria

The initial search results from each database were merged into a single file with duplicate entries removed. Subsequently, three independent reviewers screened the articles applying the inclusion criteria: (i) publication in English, and (ii) evaluation of CERAMENT™ in orthopedic surgery. Exclusion criteria included (i) absence of relevant keywords, (ii) book chapters or notes, (iii) conference abstracts or papers, (iv) purely non-human studies, (v) studies irrelevant to CERAMENT™ or orthopedic use, (vi) non-English language publications, and (vii) review articles or systematic reviews. The reason for the exclusion of each record is reported in Supplementary File S2. Titles and abstracts were independently screened by two reviewers, with disagreements resolved through consultation with a third reviewer.

## 3. Results

### 3.1. Literature Research

**Figure 1:**
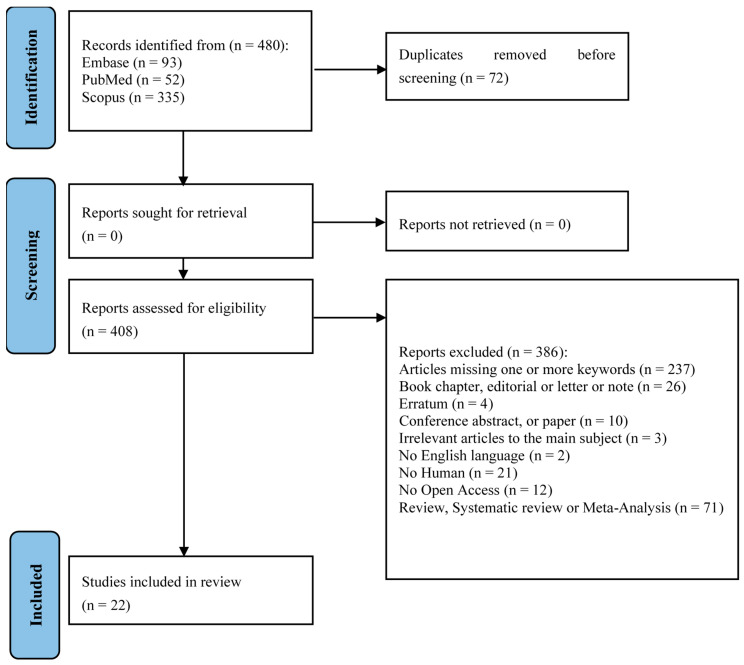
PRISMA-ScR flow-diagram showing research strategy [22].

The flowchart provides a clear illustration of the process for literature searching (Figure 1). A total of 480 articles were obtained by interrogating Embase, PubMed, and Scopus based on the following keywords: “CERAMENT™” and “Orthopedics. After removing 72 duplicates, 408 unique articles underwent title and abstract screening against predefined inclusion and exclusion criteria. Following full-text review, 22 articles were deemed relevant and included in this scoping review to elucidate the clinical applications of CERAMENT™ across various orthopedic domains. The resulting dataset provides a comprehensive foundation for synthesizing experimental outcomes, interpreting the effectiveness of CERAMENT™ in real-world settings, and drawing evidence-based conclusions regarding its use in trauma, infection, oncology, spinal, and foot and ankle surgery.

### 3.2. Study Characteristics

The key characteristics of the 22 included studies are summarized in Table 1. Between 2010 and 2025, several studies explored the applications of CERAMENT™ and related antibiotic–HA composites in orthopedic practice. All studies were conducted on human subjects. The number of participants per study ranged from 1 to 163, with a total of 1131 participants across all studies. These studies employed various methodologies, including case reports (n = 4), case series (n = 2), retrospective cohorts (n = 7), prospective cohorts (n = 7), pilot studies (n = 2), and randomized comparative trials (n = 1). Based on the outcomes of each study, we analyzed the effects of CERAMENT™ in orthopedic practice.

### 3.3. Clinical Outcome

**Figure 2:**
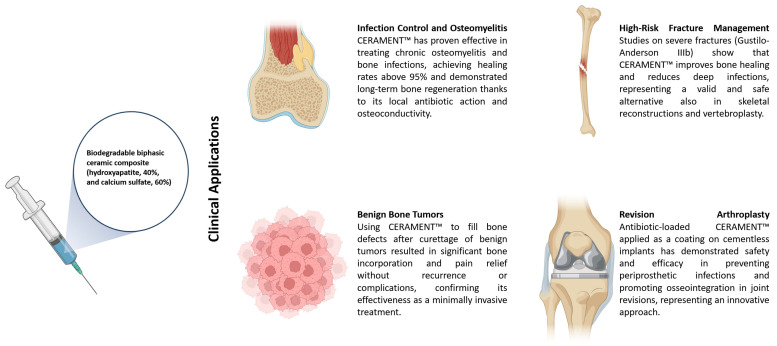
Schematic representation of CERAMENT™ applications.

CERAMENT™ emerged as a versatile solution across several orthopedic domains (Figure 2).

#### 3.3.1. Infection Control & Osteomyelitis

In a comprehensive assessment of CERAMENT™ as an antibiotic-loaded bone substitute, Anugraha et al. [17] described a 13-patient case series involving end-capping osteomyelitic lower-limb stumps, achieving 100% healing rates without infection recurrence. Similarly, Drampalos et al. [15] demonstrated that CERAMENT™ G used during debridement of chronic implant-related osteomyelitis provided excellent dead-space control and infection resolution, while Hughes LD et al. [25] introduced a novel method for coating intramedullary nails using CERAMENT™ V. This approach provided both mechanical stability and localized antibiotic release, effectively preventing biofilm formation and controlling osteomyelitis without coating debonding or adverse events—demonstrating feasibility and cost-efficiency in resource-limited settings.

A landmark study by Ferguson et al. [18] analyzed 163 cases with both radiological imaging and histological biopsies, revealing a 95.7% infection eradication rate, an average void-filling of 73.8%, and evidence of active remodeling into lamellar bone over follow-up periods extending up to nearly five years. Fraga and co-workers [14] later reported a unique case of *Mycobacterium fortuitum* osteomyelitis of the cuboid bone treated with a single-stage approach; after debridement, the defect was filled with CERAMENT™ G/V, leading to full resolution within five months.

Hoveidaei et al.’s [19] recent series of 21 patients showed a 95.2% infection clearance with a median resolution time of 128 days. Furthermore, McNally’s landmark prospective cohort of 100 chronic osteomyelitis cases reported a 96% infection eradication rate at a mean follow-up of ~19 months, with fracture and leakage rates below 6%; his six-year follow-up confirmed a sustained 94% eradication rate.

Furthermore, McNally’s landmark prospective cohort [32] of 100 chronic osteomyelitis cases reported a 96% infection eradication rate at a mean follow-up of ~19 months, with fracture and leakage rates below 6%; his six-year follow-up confirmed a sustained 94% eradication rate [21].

Niemann et al. [4] retrospectively reviewed 20 patients with corticomedullary defects, documenting clinical improvement and defect filling. Additionally, Karr et al. [20] reported the off-label use of vancomycin-impregnated CERAMENT™ in diabetic forefoot osteomyelitis, showcasing its versatility beyond standard formulations.

Collectively, these data underscore CERAMENT™’s potent local bactericidal activity, osteoconductivity, and suitability for single-stage management of complex bone infections.

#### 3.3.2. High-Risk Fracture Management

Asano et al. [23] conducted a long-term follow-up study on Gustilo–Anderson IIIb open fractures treated with antibiotic-HA CERAMENT™, reporting an impressive 96% limb salvage and bone union rate, with only 3.7% deep infection—confirming its efficacy in severe trauma cases. Building on this, Jahangir and colleagues [26] performed a prospective evaluation of similar IIIb fractures managed with local antibiotic-loaded biocomposite adjuncts, observing significantly reduced infection rates in comparison to standard care. Together, these two studies underscore CERAMENT™’s dual function: enhancing fracture healing while delivering effective infection prophylaxis in highly contaminated, high-risk trauma scenarios.

In parallel orthopedic applications, Nusselt et al. [3] through the 2014 CERTiFy trial, established a protocol contrasting CERAMENT™ with autograft in 136 tibial plateau fractures—laying essential groundwork for its use in load-bearing skeletal reconstructions.

Later, Peters et al. [33] retrospectively reviewed 25 clavicle fractures and non-unions treated with gentamicin-eluting CERAMENT™ G during ORIF, reporting uniform fracture consolidation and a favorable safety profile.

Turning to vertebral augmentation, Marcia et al. [30] published 1-year comparative data of CERAMENT™ versus PMMA in vertebroplasty, showing equivalent mechanical stability but added advantages of biodegradability and bone. This was further supported by Masala’s group [31], in a large non-randomized prospective study of osteoporotic vertebral augmentation using CERAMENT™ SpineSupport, which demonstrated consistent maintenance of vertebral structure and durable pain relief with no adjacent fractures over 12 months. Battistelli et al. [24] reported a novel case involving a C2 aneurysmal bone cyst in a young woman, treated non-surgically with a single percutaneous injection of a biphasic ceramic bone substitute (BCBS) composed of HA and CaS. At 6-month follow-up, CT imaging demonstrated complete cyst ossification, full cortical remodeling, and restoration of normal spinal canal anatomy—without neurological compromise or imaging artifacts. This case highlights BCBS vertebroplasty as a minimally invasive and effective alternative in managing complex cranio-cervical cystic lesions when conventional surgery or embolization poses significant risks. Finally, Rauschmann et al. [34] reinforced these findings in a multicenter cohort, showing restored vertebral height, significant pain reduction, and robust quality-of-life improvement over 18 months in osteoporotic compression fractures.

These results reinforce CERAMENT™’s growing role as a single-stage adjunct in challenging bone reconstruction and spinal procedures, offering a compelling alternative to traditional grafts or PMMA cements.

#### 3.3.3. Benign Bone Tumors

Kotrych et al. [28] conducted a 2018 retrospective cohort study of 33 patients with benign bone tumors treated via percutaneous or open curettage followed by injectable CERAMENT™ bone void filler. After a median follow-up of 10 months, they observed complete or near-complete graft incorporation in nearly 73% of cases within six months, alongside significant pain relief and improved functional scores—without any tumor recurrence or complications. Earlier, Kaczmarczyk et al. [27] carried out a 2015 pilot study involving 14 patients with benign bone tumors or cysts, applying minimally invasive CERAMENT™ injection. Strikingly, 100% of defects demonstrated full remodeling (Neer grade I or II) at 12 months, with no instances of postoperative infection or fracture.

In conclusion, both Kotrych et al. [28] and Kaczmarczyk et al. [27] demonstrate that injectable CERAMENT™ achieves dependable bone regeneration and defect remodeling in benign bone lesions—with high incorporation rates, pain relief, and zero reported complications—highlighting its promise as a minimally invasive alternative to traditional grafts.

#### 3.3.4. Revision Arthroplasty

In 2016, Logoluso et al. [29] conducted a prospective pilot study involving 20 patients undergoing two-stage revision arthroplasty for periprosthetic joint infection (hip n = 7, knee n = 13). They applied a gentamicin- or vancomycin-loaded CERAMENT™ G/V (60% CaS, 40% HA) as a coating on cementless implant stems. After 12 months, 95% of patients remained infection-free, with no radiographic signs of implant loosening and no adverse events attributable to the coating. This study represents the first clinical proof of concept supporting the safety and potential efficacy of using a resorbable, antibiotic-loaded bone substitute as an intraoperative implant coating to combat bacterial adhesion and biofilm formation without impairing osseointegration.

Building on this concept, McPherson et al. [7] conducted a 2022 retrospective analysis of 49 revision hip arthroplasty cases with significant acetabular defects. They evaluated the use of an antibiotic-loaded hydrogel coating on cementless femoral stems, demonstrating that it not only delivered effective structural support but also substantially reduced the incidence of periprosthetic infections, underscoring its considerable promise in complex hip revision settings.

## 4. Discussion

In this scoping review, we summarize the evidence from 22 studies regarding the use of CERAMENT™ and its antibiotic-loaded variants (e.g., CERAMENT G/V) across multiple orthopedic subspecialties. The collective data demonstrate its broad applicability—in trauma, infection control (especially chronic osteomyelitis), spinal surgery, implant-related settings, and in bone defect filling for benign lesions. The consistent reporting of high infection eradication rates (notably ~94% in long-term follow-up series) and favorable bone remodeling underscores the potential of CERAMENT™ as a versatile tool in orthopedic practice. However, the current body of evidence is limited by methodological heterogeneity, small sample sizes, and inconsistent outcome definitions. This section compares CERAMENT™ with alternative bone substitutes—primarily PerOssal^®^—and examines mechanistic properties, complication profiles, and future research priorities.

PerOssal^®^, a combination of CaS and nanocrystalline HA, is frequently used in conjunction with local antibiotics. In chronic osteomyelitis, Sambri et al. [35] reported a recurrence rate of 22.6% in 93 patients treated with PerOssal^®^, with a median follow-up of 21 months. Other series indicate similar rates (~19–23%) despite reasonable integration (89%) and usability in cavitary defects [35]. In contrast, CERAMENT™ G has demonstrated significantly lower recurrence rates in comparable cohorts. McNally et al. [32], for example, reported a 94% infection eradication rate at a mean follow-up of 6.05 years in a prospective series of 100 patients treated in a single-stage procedure. Moreover, in a cohort of 138 patients with radiographic follow-up ≥12 months, CERAMENT™ G demonstrated a mean void filling of 73.8% and an infection eradication rate of 95.7% [18]. Histological analysis confirmed progressive remodeling from osteoid to lamellar bone over a period ranging from 19 days to 2 years post-implantation [18]. These outcomes suggest more consistent long-term remodeling and infection control with CERAMENT™ compared to PerOssal^®^.

Despite promising results, CERAMENT™ is not without complications. One of the most reported adverse events is prolonged wound drainage or leakage. In the cohort by Niemann et al. [4], 50% of patients with corticomedullary defects (Cierny–Mader type III) required at least one revision, primarily due to local wound complications. Leakage rates of 6–10% have also been reported in other series. However, it remains unclear whether drainage alone correlates with reinfection or structural failure, and in many cases, it resolves without additional intervention. By contrast, PerOssal^®^, delivered as dry preformed pellets, appears to have a lower incidence of wound leakage but a higher reinfection rate [35,36,37]. These differences may reflect the distinct physical properties of the materials: CERAMENT™ is moldable and injectable, allowing for better conformity to defect geometry, while PerOssal^®^ relies on passive dissolution and may leave voids at the interface. CERAMENT™ consists of ~60% CaS (resorbable within 6–12 months) and ~40% HA (a stable osteoconductive scaffold) [5]. When combined with gentamicin or vancomycin, it offers local antibiotic delivery at concentrations sufficient to inhibit biofilms without systemic toxicity [10]. However, preclinical data confirm that its efficacy remains dependent on the quality of surgical debridement and soft-tissue management. In an animal model, residual infection persisted in cases of suboptimal debridement despite CERAMENT™ G implantation, underscoring its role as an adjunct—not a replacement—for surgical principles [38]. PerOssal^®^ similarly allows local antibiotic delivery, but sustained concentrations beyond 10 days remain uncertain, and its role in load-bearing applications is less well-supported [37]. Moreover, its higher recurrence rates in clinical series suggest limited efficacy in more aggressive or chronic infection settings [35].

In addition, the interpretation of available data is limited by several key factors. Firstly, the heterogeneity of patient cohorts and treatment protocols significantly hinders the comparability of studies [39,40]. Variations in defect size, infection chronicity, patient comorbidities, debridement techniques, and antibiotic regimens make it difficult to draw generalizable conclusions. Secondly, the overall methodological quality of the evidence is often insufficient [41]. Most available studies are small, retrospective, and monocentric, underscoring the urgent need for randomized controlled trials (RCTs) and long-term, multicenter cohort studies to validate current therapeutic strategies. Another major limitation lies in the variability of outcome definitions. Concepts such as “infection eradication”, “bone integration”, and “treatment failure” are inconsistently defined across studies, which compromises both reproducibility and the ability to make reliable comparisons [39]. Finally, there is a noticeable bias toward reporting positive outcomes, with a predominant focus on short-term results. This tendency often overlooks late complications and structural failures that may arise beyond two to three years of follow-up, thereby limiting the understanding of long-term treatment efficacy [42].

To enhance the evidence base and better guide clinical decision-making, future research must broaden and deepen the data in several interlinked areas. One pressing need is for rigorous head-to-head randomized controlled trials that compare substitutes such as CERAMENT™ G/V with alternatives like PerOssal^®^ under standardized conditions. These trials should employ consistent endpoints—such as infection recurrence, radiographic remodeling, validated patient-reported outcome measures (PROMs), and cost-effectiveness metrics—to allow a meaningful comparison. At present, although long-term data exist for CERAMENT™ G showing excellent infection-free rates over six years in chronic osteomyelitis, there is a lack of studies directly comparing it with PerOssal^®^ or other specific bone substitutes in a randomized fashion [18,21]. Another crucial direction involves mechanistic studies that link the physicochemical properties of graft materials—such as the ratio of HA to CaS, porosity, and antibiotic elution kinetics—with actual clinical outcomes. For example, elution studies have shown that biphasic ceramics with both calcium phosphate and CaS sustain vancomycin release over weeks, and higher concentrations tend to lead to higher release in early intervals [43]. Understanding how these in vitro characteristics translate into healing, infection control, remodeling, and structural integrity in vivo will help in refining materials. In parallel, refinement of surgical protocols is needed. Variables such as the extent and technique of debridement, how the defect is prepared and cleaned, timing and method of tissue and soft-tissue coverage, and postoperative care (including systemic antibiotics, dressings, weight bearing, etc.) likely play essential roles in outcomes. Evidence from severe open fractures treated with CERAMENT G suggests that when careful orthoplastic surgical technique is combined with the material, rates of limb salvage are high and deep infection rates are low, even over follow-ups of 4–5 years [23,44]. Health economic evaluations must also become more prominent. An example is the CONVICTION trial, currently underway in France, which will compare CERAMENT G plus standard treatment vs. standard treatment alone, including cost-utility (QALY-based) analysis over 24 months [41]. These studies are essential for deciding when and how to use expensive or novel substitutes in different health systems. Finally, exploration of novel indications beyond traditional chronic osteomyelitis is warranted. Off-label but carefully monitored uses in spinal reconstruction, tumor curettage, peri-implant osteolysis, or fracture augmentation hold promise. Long-term CERAMENT G studies have already shown that in complex open fractures with substantial tissue loss, the material can support bone union, limb salvage, and maintain low deep infection rates over nearly five years [41].

## 5. Conclusions

In summary, CERAMENT™ represents a significant advancement in synthetic bone grafting. Its versatility, clinical effectiveness, and safety profile position it as a valuable adjunct in the management of complex orthopedic conditions. Broader adoption into evidence-based practice will depend on continued research, clinical training, and the development of consensus guidelines.

## Figures and Tables

**Table 1:** Clinical studies of CERAMENT™ use in orthopedic applications: study design, patient details, therapeutic context, and outcomes.

Authors	Year	Study Type	Patients Enrolled	Orthopedic Application	Effect of CERAMENT™
Anugraha A et al. [17]	2020	Case series	13	End-capping of amputation stumps with chronic lower-limb osteomyelitis	All 13 healed without recurrence, no infections or hematomas
Asano E et al. [23]	2025	Retrospective cohort	76	Gustilo–Anderson IIIb open fractures with antibiotic–HA biocomposite	96% union rate, 96.3% limb salvage, 3.7% deep infection
Battistelli M et al. [24]	2024	Case report	1	C2 aneurysmal bone cyst with HA/CaS filler injection	Complete cyst resolution
Drampalos E et al. [15]	2020	Prospective cohort	52	Implant-related chronic osteomyelitis	Effective dead-space management and infection resolution
Ferguson J et al. [18]	2019	Retrospective cohort	163	Chronic osteomyelitis	95.7% infection eradication; histology confirmed lamellar bone formation
Fraga K et al. [14]	2022	Case report	1	*M. fortuitum* osteomyelitis in cuboid bone	Successful infection clearance
Hoveidaei A et al. [19]	2024	Case series	21	Chronic osteomyelitis treated with CERAMENT™ G	95.2% infection eradication, median healing in 128 days
Hughes LD et al. [25]	2019	Case Report	1	Intramedullary nail coating technique	Introduced novel CERAMENT™ V coating method, enabling local antibiotic delivery
Jahangir N et al. [26]	2019	Prospective cohort	51	Gustilo–Anderson IIIb open fractures	Lower infection rates
Kaczmarczyk J et al. [27]	2015	Pilot study	14	Benign bone tumor defect reconstruction	Complete tumor bone remodeling at 12 months
Karr J et al. [20]	2011	Case report	1	Diabetic forefoot osteomyelitis	Ulcer healed and infection resolved
Kotrych D et al. [28]	2018	Retrospective cohort	33	Benign bone tumors	Complete or near-complete graft incorporation in nearly 73% of benign bone tumor lesions
Logoluso N et al. [29]	2016	Pilot study	20	Antibiotic-loaded implant coating in arthroplasty	Reduced periprosthetic infection
Marcia S et al. [30]	2012	Prospective cohort	33	Vertebroplasty	Comparable outcomes to PMMA with osteoconductive advantages
Masala S et al. [31]	2012	Prospective cohort	80	Osteoporotic vertebral fraction augmentation	Safe and effective structural support in compression fractures
McNally M et al. [32]	2016	Prospective cohort	100	Single-stage chronic osteomyelitis	96% infection eradication at ~20 months
McNally M et al. [21]	2022	Retrospective cohort	100	Chronic osteomyelitis long-term follow-up	Sustained 94% eradication over 6 years
McPherson E et al. [7]	2022	Retrospective cohort	49	Revision hip arthroplasty with acetabular defects	Structural support and control of infection
Niemann M et al. [4]	2022	Retrospective cohort	20	Corticomedullary defects in chronic osteomyelitis	Clinical improvement and defect filling
Nusselt T et al. [3]	2014	Prospective cohort	136	Tibial plateau fractures (CERTiFy trial)	Protocol established comparing CERAMENT™ and autograft
Peters J et al. [33]	2022	Retrospective cohort	25	Clavicle fractures and non-unions	Successful bone healing
Rauschmann M et al. [34]	2010	Prospective cohort	15	Vertebral compression fracture augmentation (SpineSupport)	Pain reduction and vertebral height restoration achieved

Abbreviations: HA: hydroxyapatite; CaS: calcium sulfate; M: mycobacterium; V: vancomycin; G: gentamicin; PMMA: polymethyl methacrylate; IIIb: Gustilo–Anderson Classification Grade IIIb.

## Data Availability

All data generated or analyzed during this study are included in this published article and its Supplementary Information Files.

## Supplementary Materials

The following supporting information can be downloaded at https://www.mdpi.com/article/10.3390/jcm14217455/s1, File S1: PRISMA checklist; File S2: List of studies not included and exclusion reasons.

## Abbreviations

The following abbreviations are used in this manuscript:

CaS	calcium sulfate
HA	hydroxyapatite
MIC	minimum inhibitory concentration
PMMA	polymethyl methacrylate
PRISMA-ScR	Preferred Reporting Items for Systematic Reviews and Meta-Analyses extension for Scoping Reviews
