# Supplementary material for: A Scoping Review of CERAMENT™ Applications in Orthopedic Surgery
Source: J Clin Med. 2025 Oct 22;14(21):7455. doi: 10.3390/jcm14217455 (PMC12609670; doi:10.3390/jcm14217455)
Supplement: Supplementary file 1 [file jcm-14-07455-s001.zip › jcm-3870132-supplementary.pdf]

Supplementary File 1

Preferred Reporting Items for Systematic reviews and Meta-Analyses extension for Scoping Reviews (PRISMA-ScR) Checklist

SECTION	ITEM	PRISMA-ScR CHECKLIST ITEM	REPORTED ON PAGE #
**TITLE**			
Title	1	Identify the report as a scoping review.	1
**ABSTRACT**			
Structured summary	2	Provide a structured summary that includes (as applicable): background, objectives, eligibility criteria, sources of evidence, charting methods, results, and conclusions that relate to the review questions and objectives.	1
**INTRODUCTION**			
Rationale	3	Describe the rationale for the review in the context of what is already known. Explain why the review questions/objectives lend themselves to a scoping review approach.	1-2
Objectives	4	Provide an explicit statement of the questions and objectives being addressed with reference to their key elements (e.g., population or participants, concepts, and context) or other relevant key elements used to conceptualize the review questions and/or objectives.	2
**METHODS**			
Protocol and registration	5	Indicate whether a review protocol exists; state if and where it can be accessed (e.g., a Web address); and if available, provide registration information, including the registration number.	3
Eligibility criteria	6	Specify characteristics of the sources of evidence used as eligibility criteria (e.g., years considered, language, and publication status), and provide a rationale.	3
Information sources*	7	Describe all information sources in the search (e.g., databases with dates of coverage and contact with authors to identify additional sources), as well as the date the most recent search was executed.	3
Search	8	Present the full electronic search strategy for at least 1 database, including any limits used, such that it could be repeated.	3
Selection of sources of evidence†	9	State the process for selecting sources of evidence (i.e., screening and eligibility) included in the scoping review.	3
Data charting process‡	10	Describe the methods of charting data from the included sources of evidence (e.g., calibrated forms or forms that have been tested by the team before their use, and whether data charting was done independently or in duplicate) and any processes for obtaining and confirming data from investigators.	3
Data items	11	List and define all variables for which data were sought and any assumptions and simplifications made.	3
Critical appraisal of individual sources of evidence§	12	If done, provide a rationale for conducting a critical appraisal of included sources of evidence; describe	3

SECTION	ITEM	PRISMA-ScR CHECKLIST ITEM	REPORTED ON PAGE #
		the methods used and how this information was used in any data synthesis (if appropriate).	
Synthesis of results	13	Describe the methods of handling and summarizing the data that were charted.	3
**RESULTS**			
Selection of sources of evidence	14	Give numbers of sources of evidence screened, assessed for eligibility, and included in the review, with reasons for exclusions at each stage, ideally using a flow diagram.	4-5
Characteristics of sources of evidence	15	For each source of evidence, present characteristics for which data were charted and provide the citations.	4-8
Critical appraisal within sources of evidence	16	If done, present data on critical appraisal of included sources of evidence (see item 12).	4-8
Results of individual sources of evidence	17	For each included source of evidence, present the relevant data that were charted that relate to the review questions and objectives.	6-8
Synthesis of results	18	Summarize and/or present the charting results as they relate to the review questions and objectives.	6-8
**DISCUSSION**			
Summary of evidence	19	Summarize the main results (including an overview of concepts, themes, and types of evidence available), link to the review questions and objectives, and consider the relevance to key groups.	8-9
Limitations	20	Discuss the limitations of the scoping review process.	8-9
Conclusions	21	Provide a general interpretation of the results with respect to the review questions and objectives, as well as potential implications and/or next steps.	9
**FUNDING**			
Funding	22	Describe sources of funding for the included sources of evidence, as well as sources of funding for the scoping review. Describe the role of the funders of the scoping review.	9

JBI = Joanna Briggs Institute; PRISMA-ScR = Preferred Reporting Items for Systematic reviews and Meta-Analyses extension for Scoping Reviews.

* Where *sources of evidence* (see second footnote) are compiled from, such as bibliographic databases, social media platforms, and Web sites.

† A more inclusive/heterogeneous term used to account for the different types of evidence or data sources (e.g., quantitative and/or qualitative research, expert opinion, and policy documents) that may be eligible in a scoping review as opposed to only studies. This is not to be confused with *information sources* (see first footnote).

‡ The frameworks by Arksey and O'Malley (6) and Levac and colleagues (7) and the JBI guidance (4, 5) refer to the process of data extraction in a scoping review as data charting.

§ The process of systematically examining research evidence to assess its validity, results, and relevance before using it to inform a decision. This term is used for items 12 and 19 instead of "risk of bias" (which is more applicable to systematic reviews of interventions) to include and acknowledge the various sources of evidence that may be used in a scoping review (e.g., quantitative and/or qualitative research, expert opinion, and policy document).

From: Tricco AC, Lillie E, Zarin W, O'Brien KK, Colquhoun H, Levac D, et al. PRISMA Extension for Scoping Reviews (PRISMAScR): Checklist and Explanation. Ann Intern Med. 2018;169:467–473. doi: 10.7326/M18-0850.

Supplementary File 2. List of studies not included and exclusion reasons.

Author		Title	Year	Exclusion Criteria
Abramo A	et al.	Clinical device-related article osteotomy of distal radius fracture malunion using a fast remodeling bone substitute consisting of calcium sulphate and calcium phosphate	2010	No Open Access
Abramo A	et al.	Osteotomy of distal radius fracture malunion using a fast remodeling bone substitute consisting of calcium sulphate and calcium phosphate	2010	No Open Access
Adams B	et al.	Wrist Arthrodesis for Failed Total Wrist Arthroplasty	2016	Erratum
Ahluwalia R	et al.	Advances in pharmacotherapy for diabetic foot osteomyelitis	2021	Review
Ahmed H	et al.	Antibiotic-loaded Bone Cement in the Management of Neosternal Osteomyelitis	2022	Missing one o more keywords
Al-Fotawei R	et al.	Radiological assessment of bioengineered bone in a muscle flap for the reconstruction of critical-size mandibular defect	2014	No Human
Alfotawi R	et al.	In situ tissue engineering using an induced muscle graft to reconstruct critical size bone defect	2017	No Open Access
Alfotawi R	et al.	A novel surgical approach for the reconstruction of critical-size mandibular defects using calcium sulphate/hydroxyapatite cement, BMP-7 and mesenchymal stem cells-histological assessment	2016	No Open Access
Alfotawi R	et al.	Assessment of cellular viability on calcium sulphate/hydroxyapatite injectable scaffolds	2013	No Open Access
Aljawadi A	et al.	Adjuvant Local Antibiotic Hydroxyapatite Bio-Composite in the management of open Gustilo Anderson IIIB fractures. Prospective Review of 80 Patients from the Manchester Ortho-Plastic Unit	2020	Review
Aljawadi A	et al.	Radiological Analysis of Gentamicin Eluting Synthetic Bone Graft Substitute Used in the Management of Patients With Traumatic Bone Voids	2022	Missing one o more keywords
Alt V	et al.	Treatment of an infected nonunion with additional fresh fracture of the femur with a silver-coated intramedullary nail: A case report	2022	Missing one o more keywords
Anagnostakos K	et al.	Osteomyelitis of the First Metatarsal Head Treated With Joint-Preserving Surgery and a Synthetic Resorbable Bone Graft Substitute: A Case Report	2018	Missing one o more keywords
Anugraha A	et al.	Erratum: A novel technique for fabricating antibiotic-coated intramedullary nails using an antibiotic-loaded calcium sulphate hydroxyapatite bio-composite, Cerament-V (Journal of Surgical Case Reports DOI: 10.1093/jscr/rjz327)	2020	Erratum
Arabmotlagh M	et al.	[Füllmaterialien zur Augmentation von osteoporotischen Wirbelkörperfrakturen]"	2010	No English
Arcos D	et al.	The relevance of biomaterials to the prevention and treatment of osteoporosis	2014	Review
Armbruster J	et al.	Treatment of High-Grade Chronic Osteomyelitis and Nonunions with PerOssal®: A Retrospective Analysis of Clinical Efficacy and Patient Perspectives	2024	Missing one o more keywords
Arnold J	et al.	Characteristics of postoperative weight bearing and management protocols for tibial plateau fractures: Findings from a scoping review	2017	Review
Arrigoni F	et al.	Developments in the management of bone metastases with interventional radiology	2018	Review
Asadi-Eydivand M	et al.	Structure, properties, and in vitro behavior of heat-treated calcium sulfate scaffolds fabricated by 3D printing	2016	Missing one o more keywords
Axelrod D	et al.	Proximal Tibia Fractures	2021	Book Chapter
Badman B	et al.	A biomechanical analysis of flowable injectable calcium bone void filler on acromial tensile stresses: a method to reduce acromial stress fractures	2021	No Open Access
Banche-Niclot F	et al.	Optimization of an Injectable, Resorbable, Bioactive Cement Able to Release the Anti-Osteoclastogenic Biomolecule ICOS-Fc for the Treatment of Osteoporotic Vertebral Compression Fracture	2023	Missing one o more keywords

Bardají S	et al.	[Nous desenvolupaments en materials per vertebroplàstia]	2018	No English
Bardyugov P	et al.	Bone Defect Replacement in Diabetic Neuropathic Osteoarthropathy (Charcot Foot): Review	2023	Review
Barker J	et al.	A formidable foe is sabotaging your results: What you should know about biofilms and wound healing	2017	Missing one o more keywords
Bartyzel A	et al.	Treatment of massive giant cell tumor of bone of the proximal radius. Case study	2019	No Open Access
Basaran S	et al.	Partial load-bearing rabbit ulnar segmental defects are regenerated with biocompatible grafts with or without bone marrow-derived mesenchymal stem cells	2022	No Human
Berger C	et al.	High Risk for Persistent Peri-Prosthetic Infection and Amputation in Mega-Prosthesis Reconstruction	2023	Missing one o more keywords
Bezstarosti H	et al.	In Vitro Elution of Gentamicin from CERAMENT® G Has an Antimicrobial Effect on Bacteria With Various Levels of Gentamicin Resistance Found in Fracture-related Infection	2024	No Human
Bhaskaran N	et al.	Nanotherapeutics in the management of bone infections	2025	Book Chapter
Bhatti P	et al.	Multidisciplinary Approach to a Transverse Periprosthetic Femur Fracture in a Short-Statured Patient: A Case Report	2025	Missing one o more keywords
Bidossi A	et al.	In vitro evaluation of gentamicin or vancomycin containing bone graft substitute in the prevention of orthopedic implant-related infections	2020	Missing one o more keywords
Billström G	et al.	Application of scaffolds for bone regeneration strategies: Current trends and future directions	2013	Missing one o more keywords
Blirup-Plum S	et al.	Pathological and microbiological impact of a gentamicin-loaded biocomposite following limited or extensive debridement in a porcine model of osteomyelitis	2020	No Human
Bohner M	et al.	Design of ceramic-based cements and putties for bone graft substitution	2010	Missing one o more keywords
Borens O	et al.	[Infizierte Osteosynthese]"	2016	Review
Boriani S	et al.	Aneurysmal bone cyst of C2 treated with novel anterior reconstruction and stabilization	2019	Note
Bostelmann R	et al.	First report on treating spontaneous infectious spondylodiscitis of lumbar spine with posterior debridement, posterior instrumentation and an injectable calcium sulfate/hydroxyapatite composite eluting gentamicin: a case report	2016	No Open Access
Boyle K	et al.	Vancomycin-laden calcium phosphate-calcium sulfate composite allows bone formation in a rat infection model	2019	No Human
Bruno F	et al.	Weight-bearing MRI of the knee: A review of advantages and limits	2018	Review
Butini M	et al.	In vitro anti-biofilm activity of a biphasic gentamicin-loaded calcium sulfate/hydroxyapatite bone graft substitute	2018	No Human
Bydon M	et al.	Evaluation and Treatment of Osteoporotic Fractures (Cement Augmentation)	2023	Book Chapter
Cao S	et al.	New perspectives: In-situ tissue engineering for bone repair scaffold	2020	Review
Caracchini G	et al.	Antibiotic Containing Bone Substitute in Major Hip Surgery: A Long Term Gentamicin Elution Study	2018	Missing one o more keywords
Caranci F	et al.	Magnetic resonance imaging correlates of benign and malignant alterations of the spinal bone marrow	2018	Missing one o more keywords
Cazzato R	et al.	French Multidisciplinary Approach for the Treatment of MSK Tumors	2020	Missing one o more keywords
Chan K	et al.	In vivo evaluation of a new β-tricalcium phosphate bone substitute in a rabbit femur defect model	2015	Missing one o more keywords

Chatzipapas C	et al.	Local Antibiotic Delivery Systems in the Surgical Treatment of Diabetic Foot Osteomyelitis: Again	2022	Missing one o more keywords
Chen B	et al.	The implantation of a Nickel-Titanium shape memory alloy ameliorates vertebral body compression fractures: A cadaveric study	2015	Missing one o more keywords
Chen H	et al.	Evaluation of Antibiotic-Loaded Bone Cement in Treatment of Infected Diabetic Foot: Systematic Review and Meta-Analysis	2024	Missing one o more keywords
Chen H	et al.	Recent progress in the diagnosis and treatment of posterior tibial plateau fractures	2015	Missing one o more keywords
Chen P	et al.	Devascularized Bone Surface Culture: A Novel Strategy for Identifying Osteomyelitis-Related Pathogens	2022	Missing one o more keywords
Chen Y	et al.	A novel antibacterial zirconia-containing PMMA bone cement	2022	Missing one o more keywords
Chenbing Z	et al.	Research progress of antibiotic loaded bone cement and its application in treating diabetic foot infected wounds	2022	Review
Chiang C	et al.	An assessment of physical properties and the viability of osteoblast-like cells of cefazolin-impregnated calcium sulfate bone-void filler	2023	Missing one o more keywords
Chiang J	et al.	A Toolbox of Bone Consolidation for the Interventional Radiologist	2023	Review
Cianni L	et al.	Predictive Factors of Poor Outcome in Sanders Type III and IV Calcaneal Fractures Treated with an Open Reduction and Internal Fixation with Plate: A Medium-Term Follow-Up	2022	Missing one o more keywords
Clark A	et al.	A Two-Stage Approach Integrating Provisional Biomaterial-Mediated Stabilization Followed by a Definitive Treatment for Managing Volumetric Muscle Loss Injuries	2024	Missing one o more keywords
Colding-Rasmussen T	et al.	Antibiotic Elution Characteristics and Pharmacokinetics of Gentamicin and Vancomycin from a Mineral Antibiotic Carrier: An in vivo Evaluation of 32 Clinical Cases	2018	Missing one o more keywords
Colon D	et al.	Assessment of the injection behavior of commercially available bone BSMs for Subchondroplasty® procedures	2015	Missing one o more keywords
Compagnoni R	et al.	Arthroscopic-assisted reduction and fixation of proximal tibial fractures: Personal surgical technique	2021	Missing one o more keywords
Cook E	et al.	Bone Graft Substitutes and Allografts for Reconstruction of the Foot and Ankle	2009	Review
Cooper G	et al.	Autologous versus synthetic bone grafts for the surgical management of tibial plateau fractures: a systematic review and meta-analysis of randomized controlled trials	2022	Missing one o more keywords
Corona P	et al.	Uniplanar versus biplanar monolateral external fixator knee arthrodesis after end-stage failed infected total knee arthroplasty: a comparative study	2020	Missing one o more keywords
Cortez P	et al.	A glass-reinforced hydroxyapatite and surgical-grade calcium sulfate for bone regeneration: In vivo biological behavior in a sheep model	2012	No Human
Cutler H	et al.	Outcome Instruments in Spinal Trauma Surgery: A Bibliometric Analysis	2016	Review
D'Aprile P	et al.	Magnetic Resonance Imaging in degenerative disease of the lumbar spine: Fat Saturation technique and contrast medium	2018	Missing one o more keywords
Dadkhah M	et al.	Preparation and characterisation of an innovative injectable calcium sulphate based bone cement for vertebroplasty application	2017	No Open Access
Das C	et al.	Role of Augmentation in the Fixation of Osteoporotic Fractures	2025	Review
Davies M	et al.	Assessing and managing open fractures: A systematic approach	2022	Missing one o more keywords

De Filippo M	et al.	Radiofrequency ablation of osteoid osteoma	2018	Review
De Pace R	et al.	Bone Regeneration: A Review of Current Treatment Strategies	2025	Review
Dong C	et al.	Percutaneous cyst aspiration with injection of two different bioresorbable bone cements in treatment of simple bone cyst	2020	Missing one o more keywords
Dong Z	et al.	Silk fibroin hydrogels induced and reinforced by acidic calcium phosphate – A simple way of producing bioactive and drug-loadable composites for biomedical applications	2021	Missing one o more keywords
Doorgakant A	et al.	An Approach to Managing Midfoot Charcot Deformities	2020	Review
Döring K	et al.	Treatment of benign bone lesions with an injectable biphasic bone substitute	2024	Missing one o more keywords
Dorozhkin S	et al.	Calcium Orthophosphate (CaPO4) Containing Composites for Biomedical Applications: Formulations, Properties, and Applications	2024	Review
Dorozhkin S	et al.	Calcium orthophosphate-containing biocomposites and their biomedical applications	2017	Book Chapter
Dorozhkin S	et al.	Hydroxyapatite and other calcium orthophosphates: Biocomposites, self-setting formulations and dissolution	2017	Book Chapter
Dorozhkin S	et al.	Calcium Orthophosphate-Based Bioceramics and Biocomposites	2016	Book Chapter
Dorozhkin S	et al.	Calcium orthophosphates: Applications in nature, biology, and medicine	2012	Book Chapter
Down B	et al.	Single-stage orthoplastic treatment of complex calcaneal osteomyelitis with large soft-tissue defects LONG-TERM FOLLOW-UP	2024	Missing one o more keywords
Dragani M	et al.	Kyphoplasty in spinal fractures with resin-like material â€œpMMAâ€ and biological material â€œcERAMENTâ€: Differences	2011	Conference Abstract
Ducloux R	et al.	Limb salvage attempt through calcaneal osteitis curettage and antibiotic impregnated cement	2016	Missing one o more keywords
Duta L	et al.	Editorial: Calcium phosphates of synthetic and natural origin: current status and future challenges	2023	Editorial
Dvorzhinskiy A	et al.	Ceramic composite with gentamicin decreases persistent infection and increases bone formation in a rat model of debrided osteomyelitis	2021	No Human
Eikani C	et al.	The State of Local Antibiotic Use in Orthopedic Trauma	2024	Review
Elhessy A	et al.	Intramedullary Canal Injection of Vancomycin-and Tobramycin-loaded Calcium Sulfate: A Novel Technique for the Treatment of Chronic Intramedullary Osteomyelitis	2022	Missing one o more keywords
Ene R	et al.	Review of Calcium-Sulphate-Based Ceramics and Synthetic Bone Substitutes Used for Antibiotic Delivery in PJI and Osteomyelitis Treatment	2021	Review
Ene R	et al.	Review of Calcium-Sulphate-Based Ceramics and Synthetic Bone Substitutes Used for Antibiotic Delivery in PJI and Osteomyelitis Treatment	2021	Review
Epstein G	et al.	Dead space management strategies in the treatment of chronic osteomyelitis: a retrospective review	2023	Review
Ercan H	et al.	Clinical Applications of Injectable Biomaterials	2018	Book Chapter
Everhart J	et al.	Depot antibiotics	2015	Review
Fahed R	et al.	Just a drop of cement: A case of cervical spine bone aneurysmal cyst successfully treated by percutaneous injection of a small amount of polymethyl-methacrylate cement	2014	Missing one o more keywords
Feng S	et al.	Implantation of an empty polyetheretherketone cage in anterior cervical discectomy and fusion: a prospective randomised controlled study with 2Â years follow-up	2018	Missing one o more keywords
Ferguson J	et al.	A comparison of clinical and radiological outcomes between two different biodegradable local antibiotic carriers used in the single-stage surgical management of long bone osteomyelitis	2023	Missing one o more keywords

Ferreira N	et al.	The role of bioceramics in the management of osteomyelitic voids	2023	No Open Access
Filippiadis D	et al.	Percutaneous Microwave Ablation and Osteoplasty of an Aneurysmal Bone Cyst	2021	Letter
Filippiadis D	et al.	Percutaneous Vertebroplasty and Kyphoplasty: Current Status, New Developments and Old Controversies	2017	Review
Fischetti T	et al.	3d printing and bioprinting to model bone cancer: The role of materials and nanoscale cues in directing cell behavior	2021	Review
Fonkoue L	et al.	The Microbiological Profile and Antibiotic Susceptibility of Fracture Related Infections in a Low Resource Setting Differ from High Resource Settings: A Cohort Study from Cameroon	2024	Missing one o more keywords
Frassanito P	et al.	Multimodal Management of Aggressive Recurrent Aneurysmal Bone Cyst of Spine: Case Report and Review of Literature	2019	Missing one o more keywords
Freischmidt H	et al.	Individualized techniques of implant coating with an antibiotic-loaded	2020	No Human
Freischmidt H	et al.	Efficacy of an Antibiotic Loaded Ceramic-Based Bone Graft Substitute for the Treatment of Infected Non-Unions	2022	Missing one o more keywords
Freischmidt H	et al.	Augmentation in surgical sepsis: Chances and limitations in the treatment of osteitis with calcium hydroxyapatite containing antibiotics	2022	Review
Freischmidt H	et al.	Individualized Techniques of Implant Coating with an Antibiotic-Loaded, Hydroxyapatite/Calcium Sulphate Bone Graft Substitute	2020	No Human
Freischmidt H	et al.	Systemic Administration of PTH Supports Vascularization in Segmental Bone Defects Filled with Ceramic-Based Bone Graft Substitute	2021	No Human
Gagala J	et al.	Minimum 10 years clinical and radiological outcomes of acetabular revisions of total hip arthroplasties with tricalcium phosphate/hydroxyapatite bone graft substitute	2021	Missing one o more keywords
Ganey T	et al.	Considerations for a physics-based	2010	Conference Abstract
Gasbarra E	et al.	Surgical treatment of tibial plateau fractures with the addition of Ceramentâ„¢ injectable bone substitute	2011	Conference Abstract
Gauzit Amiel A	et al.	Designed sponges based on chitosan and cyclodextrin polymer for a local release of ciprofloxacin in diabetic foot infections	2020	Missing one o more keywords
Geurts J	et al.	Use of contemporary biomaterials in chronic osteomyelitis treatment: Clinical lessons learned and literature review	2021	Review
Giannotti S	et al.	Angular stable plates in proximal meta-epiphyseal tibial fractures: study of joint restoration and clinical and functional evaluation	2016	Missing one o more keywords
Glaeser J	et al.	Electrospun synthetic bone void filler promotes human MSC function and BMP-2 mediated spinal fusion	2020	Missing one o more keywords
Gould M	et al.	Fever in a patient with osteomyelitis: The diagnosis could be serotonin syndrome	2021	Missing one o more keywords
Graff C	et al.	Antibiotic impregnated cement coated intramedullary nail (ACCIN) using bronchoscopy tubing: technical tips, case series and a review of the literature	2024	Review
Gragnano E	et al.	Treatment of Spinal Aneurysmal Bone Cyst with Percutaneous Injection of Hydroxyapatite Osteoconductive Cement	2023	Missing one o more keywords
Gramlich Y	et al.	Local Administration of Antibiotics in Orthopedics and Traumatology	2023	Review
Griauzde J	et al.	Successful treatment of a musculoskeletal tumor society grade 3 aneurysmal bone cyst with N-butyl cyanoacrylate embolization and percutaneous cryoablation	2015	Missing one o more keywords
Gritsch L	et al.	Tailored therapeutic release from polycaprolactone-silica hybrids for the treatment of osteomyelitis: antibiotic rifampicin and osteogenic silicates	2022	Missing one o more keywords

Guarnieri G	et al.	Update of vertebral cementoplasty in porotic patients	2015	Missing one o more keywords
Guarnieri G	et al.	Percutaneous treatment of symptomatic aneurysmal bone cyst of L5 by percutaneous injection of osteoconductive material (Cerament).	2013	Review
Güleçyüz M	et al.	The Primary Stability of a Bioabsorbable Poly-L-Lactic Acid Suture Anchor for Rotator Cuff Repair Is Not Improved with Polymethylmethacrylate or Bioabsorbable Bone Cement Augmentation	2018	Irrelevant
Guo J	et al.	Focal issues in and strategies for treatment of periarticular osteoporotic fractures	2020	Missing one o more keywords
Hammad Y	et al.	Chronic osteomyelitis of the tibia in a runner: Catastrophic consequences of shin splints	2018	Missing one o more keywords
Hammouche S	et al.	Calcium salts bone regeneration scaffolds: A review article	2012	Review
Hatten H	et al.	Bone healing using a bi-phasic ceramic bone substitute demonstrated in human vertebroplasty and with histology in a rabbit cancellous bone defect model	2012	Missing one o more keywords
HeiÃŸ C	et al.	Clinical use of bone substitutes	2013	Review
Henry JA	et al.	Long-Term Follow-Up of Open Gustilo-Anderson IIIB Fractures Treated With an Adjuvant Local Antibiotic Hydroxyapatite Bio-Composite	2023	Missing one o more keywords
Hoelscher-Doht S	et al.	Bone filler and adhesive at the same time: in-vitro analysis in a porcine fracture model	2025	Missing one o more keywords
Hofmann A	et al.	Autologous Iliac Bone Graft Compared with Biphasic Hydroxyapatite and Calcium Sulfate Cement for the Treatment of Bone Defects in Tibial Plateau Fractures: A Prospective	2020	Missing one o more keywords
Hoque M	et al.	Natural fiber-based green composites: Processing, properties and biomedical applications	2021	Missing one o more keywords
Horstmann PF	et al.	Early Clinical and Radiological Experience with a Ceramic Bone Graft Substitute in the Treatment of Benign and Borderline Bone Lesions	2018	Missing one o more keywords
Horstmann PF	et al.	Natural Course of Local Bone Mineralization After Treatment of Benign or Borderline Bone Tumors and Cysts With a Composite Ceramic Bone Graft Substitute	2018	Missing one o more keywords
Horstmann PF	et al.	Composite biomaterial as a carrier for bone-Active substances for metaphyseal tibial bone defect reconstruction in rats	2017	Missing one o more keywords
Hossain M	et al.	A composite of polymethylmethacrylate, hydroxyapatite, and β-tricalcium phosphate for bone regeneration in an osteoporotic rat model	2023	Missing one o more keywords
Hotchen A	et al.	Can we predict outcome after treatment of long bone osteomyelitis? A study of pAtient-reported quAlity of life strAtified with the BACh classification	2020	Missing one o more keywords
Hou L	et al.	The application of biodegradable absorbable materials in bone repair	2016	Missing one o more keywords
Hoveidaei AH	et al.	Local antibiotic delivery: Recent basic and translational science insights in orthopedics	2025	Review
Hoveidaei AH	et al.	The Efficacy of Calcium Sulfate/Hydroxyapatite (CaS/HA) Gentamicin in Osteomyelitis Treatment: A Case Series	2024	Missing one o more keywords
Howard T	et al.	Treatment of Infected Nonunions With Bone Defects Using Autologous Bone Graft and Absorbable Antibiotic-Loaded Calcium Sulfate–Hydroxyapatite Paste	2022	Missing one o more keywords

Hsieh C	et al.	Leiomyoma of the Ankle	2024	Missing one o more keywords
Hsieh D	et al.	Supercritical Carbon Dioxide-derived Bone Graft Putty: Regenerative Efficacy In Rabbit Bone Defect Model	2023	Conference Abstract
Huang J	et al.	A calcium sulphate/hydroxyapatite ceramic biomaterial carrier for local delivery of tobramycin in bone infections: Analysis of rheology, drug release and antimicrobial efficacy	2023	Missing one o more keywords
Huang T	et al.	Outcomes and second-look arthroscopic evaluation after combined arthroscopic treatment of tibial plateau and tibial eminence avulsion fractures: A 5-year minimal follow-up Orthopedics and biomechanics	2015	Missing one o more keywords
Hung P	et al.	Preoperative planning of compact zone trajectory is necessary in treating osteoporotic vertebral compression fracture with endplate involvement: A prospective randomized controlled study	2023	Missing one o more keywords
Ipponi E	et al.	Fixation with Carbon Fiber Plates After Curettage in Benign and Locally Aggressive Bone Tumors: Clinical and Radiographic Outcomes	2025	Missing one o more keywords
Irmola T	et al.	Solitary juvenile xanthogranuloma in the spine pretreated with neoadjuvant denosumab therapy followed by surgical resection in a 5-year-old child: case report and literature review	2018	Review
Ismat A	et al.	Antibiotic cement coating in orthopedic surgery: a systematic review of reported clinical techniques	2021	Review
Iundusi R	et al.	Augmentation of tibial plateau fractures with an injectable bone substitute: CERAMENT(â„¢). Three year follow-up from a prospective study	2015	Review
Jacobs A	et al.	Biological properties of copper-doped biomaterials for orthopedic applications: A review of antibacterial angiogenic and osteogenic aspects	2020	Review
Jensen L	et al.	Intraoperative tissue sampling for histology in chronic osteomyelitis shows high neutrophil infiltration centrally and low remains in debrided presumed infection-free regions	2024	Missing one o more keywords
Jia P	et al.	Prophylactic vertebroplasty procedure applied with a resorbable bone cement can decrease the fracture risk of sandwich vertebrae: Long-term evaluation of clinical outcomes	2017	Missing one o more keywords
Jiang X	et al.	Limb salvage and prevention of ulcer recurrence in a chronic refractory diabetic foot osteomyelitis	2020	Missing one o more keywords
Jinno Y	et al.	Impact of surface contamination of implants with saliva during placement in augmented bone defects in sheep calvaria	2019	No Human
Kalantar S	et al.	Marginal bone resection and immediate internal fixation in multidrug resistant chronic septic nonunions of lower limb long bones: a case series	2025	Missing one o more keywords
Kankilic B	et al.	Apatites for orthopedic applications	2017	Book Chapter
Karakoyun D	et al.	Vancomycin-laden calcium phosphate-calcium sulfate composite allows bone formation in a rat infection model	2021	No Human
Karr J	et al.	Improving outcomes for osteomyelitis after partial bone resection	2021	Missing one o more keywords
Karr J	et al.	Lower-extremity osteomyelitis treatment using calcium sulfate/hydroxyapatite bone void filler with antibiotics seven-year retrospective study	2018	Missing one o more keywords
Karr J	et al.	An overview of the percutaneous antibiotic delivery technique for osteomyelitis treatment and a case study of calcaneal osteomyelitis	2017	Review
Karr J	et al.	In vitro antimicrobial activity of calcium sulfate and hydroxyapatite (Cerament Bone Void Filler) discs using heat-sensitive and non-heat-sensitive antibiotics against methicillin-resistant Staphylococcus aureus and Pseudomonas aeruginosa	2011	No Human
Kavarthapu V	et al.	Treatment of High-Grade Chronic Osteomyelitis and Nonunions with PerOssal®: A Retrospective Analysis of Clinical Efficacy and Patient Perspectives	2024	Missing one o more keywords

Kavarthapu V	et al.	Two-stage reconstruction of infected Charcot foot using internal fixation A Promising Functional Limb Salvage Technique	2021	Missing one o more keywords
Kavarthapu V	et al.	Evaluation of Adjuvant Antibiotic Loaded Injectable Bio-Composite Material in Diabetic Foot Osteomyelitis and Charcot Foot Reconstruction	2023	Missing one o more keywords
Kelekis A	et al.	Vertebroplasty and kyphoplasty in malignant vertebral fracture	2013	Book Chapter
Keppler A	et al.	Bone defect reconstruction with a novel biomaterial containing calcium phosphate and aluminum oxide reinforcement	2020	Missing one o more keywords
Kevin A	et al.	Effect of pedicle fill on axial pullout strength in spinal fixation after rod reduction	2017	Missing one o more keywords
Khan H	et al.	The use of bioabsorbable materials in orthopaedics	2021	Missing one o more keywords
Khansa I	et al.	Use of antibiotic impregnated resorbable beads reduces pressure ulcer recurrence: A retrospective analysis	2018	Missing one o more keywords
Khayatan D	et al.	Cross Talk Between Cells and the Current Bioceramics in Bone Regeneration: A Comprehensive Review"	2024	Review
Kjellberg A	et al.	A novel treatment strategy with hyperbaric oxygen of chronic osteomyelitis and pseudoarthrosis in a child with congenital hereditary sensory and autonomic neuropathy type 4 congenital insensitivity to pain with anhidrosis syndrome: a case report	2025	Missing one o more keywords
Kleber C	et al.	Complication management of infected osteosynthesis: Therapy algorithm for peri-implant infections	2013	Missing one o more keywords
Kobbe P	et al.	Convergence of scaffold-guided bone regeneration and RIA bone grafting for the treatment of a critical-sized bone defect of the femoral shaft	2020	Missing one o more keywords
Koch G	et al.	Percutaneous Treatments of Benign Bone Tumors	2018	Missing one o more keywords
Kok J	et al.	Augmenting a dynamic hip screw with a calcium sulfate/hydroxyapatite biomaterial	2021	Missing one o more keywords
Kok J	et al.	Fracture strength of the proximal femur injected with a calcium sulfate/hydroxyapatite bone substitute	2019	Missing one o more keywords
Kotrych D	et al.	Preliminary Results of Surgical Treatment for Enchondroma Using a Novel Bioactive and Osseoconductive HAP/β–Glucan Bone Substitute FlexiOss®—Case Series	2025	Missing one o more keywords
Kotsarinis G	et al.	Stabilization of Tibial Fractures at Risk of Complications with the Bactiguard Intramedullary Nail: Early to Medium Results with a Novel Metal-Coated Device	2023	Missing one o more keywords
Kubosch E	et al.	Clinical trial and in-vitro study comparing the efficacy of treating bony lesions with allografts versus synthetic or highly-processed xenogeneic bone grafts	2016	Missing one o more keywords
Kulkarni S	et al.	Treatment of a spinal aneurysmal bone cyst using combined image-guided cryoablation and cementoplasty	2015	Missing one o more keywords
Kumar B	et al.	Aneurysmal bone cyst of thoracic spine with neurological deficit and its recurrence treated with multimodal intervention - A case report	2020	Missing one o more keywords
Lakshmi Priya M	et al.	Ceramic nanofiber composites	2017	Book Chapter

Lari A	et al.	Correction to: Single versus two-stage management of long-bone chronic osteomyelitis in adults: a systematic review and meta-analysis (Journal of Orthopaedic Surgery and Research, (2024), 19, 1, (351), 10.1186/s13018-024-04832-7)	2024	Erratum
Laubach M	et al.	Clinical translation of a patient-specific scaffold-guided bone regeneration concept in four cases with large long bone defects	2022	Missing one o more keywords
Laycock P	et al.	Effects of antibiotic addition on the setting time of calcium sulphate bone cement	2011	Conference Paper
Le Ferrec M	et al.	Design and properties of a novel radiopaque injectable apatitic calcium phosphate cement, suitable for image-guided implantation	2018	Missing one o more keywords
Leet A	et al.	Bone-grafting in polyostotic fibrous dysplasia	2016	Missing one o more keywords
Lessing N	et al.	Complex lumbar spine fusion for an elderly patient under spinal anesthesia	2017	Missing one o more keywords
Lewis G	et al.	Viscoelastic properties of injectable bone cements for orthopaedic applications: State-of-the-art review	2011	Review
Li J	et al.	A Novel Approach for Percutaneous Vertebroplasty Based on Preoperative Computed Tomography–Based Three-Dimensional Model Design	2017	Missing one o more keywords
Liang J	et al.	Arthroscopic-assisted inflatable bone tamp reduction for treatment of posterolateral tibial plateau fractures	2018	Missing one o more keywords
Liao G	et al.	Effect of methotrexate on the mechanical properties and microstructure of calcium phosphate cement	2014	Missing one o more keywords
Liguori A	et al.	Clinical and instrumental assessment of herniated discs after nucleoplasty: A preliminary study	2014	Missing one o more keywords
Lin D	et al.	Fabrication and clinical application of easy-to-operate pre-cured CPC/rhBMP-2 micro-scaffolds for bone regeneration	2016	Missing one o more keywords
Lin J	et al.	Distribution of bone voids in the thoracolumbar spine in Chinese adults with and without osteoporosis: A cross-sectional multi-center study based on 464 vertebrae	2023	Missing one o more keywords
Linhart C	et al.	Micro-Structural and Biomechanical Evaluation of Bioresorbable and Conventional Bone Cements for Augmentation of the Proximal Femoral Nail	2023	Missing one o more keywords
Liodaki E	et al.	The Use of Bone Graft Substitute in Hand Surgery: A Prospective Observational Study	2016	Erratum
Liu J	et al.	Coated nails: is their use supported by the literature?	2021	Conference Paper
Liu X	et al.	Finite Element Analysis and Clinical Application of Unilateral/Bilateral Percutaneous Vertebroplasty for Treating Osteoporotic Vertebral Compression Fractures in Elderly Patients	2018	Missing one o more keywords
Liu Y	et al.	Longitudinal in vivo biodistribution of nano and micro sized hydroxyapatite particles implanted in a bone defect	2022	Missing one o more keywords
Liu Z	et al.	Comparison of outcomes following tirobot-assisted sacroiliac screw fixation with bone grafting and traditional screw fixation without bone grafting for unstable osteoporotic sacral fracture: A single-center retrospective study of 33 patients	2021	Missing one o more keywords
Lopas L	et al.	Outcomes of Various Antibiotic Cement-Coated Intramedullary Implants on the Treatment of Long Bone Septic Nonunion	2022	Missing one o more keywords
Lorentzen A	et al.	One-stage treatment of chronic osteomyelitis with an antibiotic-loaded biocomposite and a local or free flap	2021	Missing one o more keywords

Lukina Y	et al.	Chemically Bound Resorbable Ceramics as an Antibiotic Delivery System in the Treatment of Purulent–Septic Inflammation of Bone Tissue	2022	No Human
Lun D	et al.	Limitations and modifications in the clinical application of calcium sulfate	2023	Missing one o more keywords
Lun D	et al.	Biomechanical study of injectable calcium sulfate cement	2020	Missing one o more keywords
Luo S	et al.	Combination therapy with vancomycin-loaded calcium sulfate and vancomycin-loaded PMMA in the treatment of chronic osteomyelitis	2016	Missing one o more keywords
Luo S	et al.	Distally based sural flaps for soft tissue defects following traumatic osteomyelitis of lower leg and foot	2024	Missing one o more keywords
Mair O	et al.	The Use of a Vancomycin-Eluting Calcium Sulfate and Hydroxyapatite Composite for Dead Space Management in a Fracture-Related Infection (FRI): A Retrospective Case Series	2024	Missing one o more keywords
Malhotra R	et al.	Alternating Layers of Morselized Allograft and Injectable Ceramic Bone Graft Substitute in Acetabular Reconstruction: A Novel ‘Sandwich’ Technique	2023	Missing one o more keywords
Manchikanti L	et al.	Development of an interventional pain management specific instrument for methodologic quality assessment of nonrandomized studies of interventional techniques	2014	Missing one o more keywords
Manfrè L	et al.	Vertebroplasty and spinal tumors	2013	Book Chapter
Mao Y	et al.	No Superior Bone Union Outcomes with Allografts Compared to No Grafts and Autografts Following Medial Opening Wedge High Tibial Osteotomy: A Retrospective Cohort Study	2024	Missing one o more keywords
Mao Y	et al.	Lack of Efficacy of Bone Void Filling Materials in Medial Opening-Wedge High Tibial Osteotomy: A Systematic Review and Network Meta-analysis	2023	Review
Marcia S	et al.	Percutaneous stabilization of lumbar spine: A literature review and new options in treating spine pain	2016	Review
Marczak D	et al.	The use of calcium carbonate beads containing gentamicin in the second stage septic revision of total knee arthroplasty reduces reinfection rate	2016	Missing one o more keywords
Markakis K	et al.	Local Antibiotic Delivery Systems: Current and Future Applications for Diabetic Foot Infections	2018	Missing one o more keywords
Masala S	et al.	Treatment of painful Modic type I changes by vertebral augmentation with bioactive resorbable bone cement	2014	Missing one o more keywords
Masala S	et al.	Thoraco-lumbar traumatic vertebral fractures augmentation by osteo-conductive and osteo-inductive bone substitute containing strontium-hydroxyapatite: Our experience	2014	Missing one o more keywords
Mastan S	et al.	Novel reconstruction of an open pilon fracture with significant bone loss	2019	Missing one o more keywords
McNally M	et al.	Comment on Niemann et al. Outcome Analysis of the Use of Cerament® in Patients with Chronic Osteomyelitis and Corticomedullary Defects. Diagnostics 2022	2022	Letter
Mcnamara I	et al.	Surgical fixation methods for tibial plateau fractures	2017	Missing one o more keywords
Michelini G	et al.	Dynamic MRI in the evaluation of the spine: State of the art	2019	Review
Mifsud M	et al.	Paediatric bone and joint infections: a guide from diagnosis to management	2023	Missing one o more keywords
Mintz D	et al.	Bone Tumor Imaging, Then and Now: Review Article	2014	Review

Modi Y	et al.	Process parameter optimization for porosity and compressive strength of calcium sulfate based 3D printed porous bone scaffolds	2021	Missing one o more keywords
Moussi H	et al.	Injectable macromolecule-based calcium phosphate bone substitutes	2022	Review
Multani I	et al.	Long-term follow-up of the use of a synthetic bone graft composite in the surgical management of primary bone tumors	2018	Missing one o more keywords
Mundis G	et al.	Why are DBMs so variable? Influence of fibers carrier and tissue bank	2020	Conference Abstract
Muto M	et al.	What's new in vertebral cementoplasty?	2016	Review
Nabet A	et al.	Survival and Outcomes of 1.5-Stage vs 2-Stage Exchange Total Knee Arthroplasty Following Prosthetic Joint Infection	2022	Missing one o more keywords
Nair M	et al.	Antibiotic releasing biodegradable scaffolds for osteomyelitis	2014	Missing one o more keywords
Nazarova N	et al.	The surgical management of the cavity and bone defects in enchondroma cases: A prospective randomized trial	2021	Missing one o more keywords
Nezwek T	et al.	Acute prophylactic antibiotic nailing of open femoral shaft fractures for prevention of fracture-related infection	2024	Missing one o more keywords
Niazi NS	et al.	Adjuvant antibiotic loaded bio composite in the management of diabetic foot osteomyelitis — A multicentre study	2019	Missing one o more keywords
Nicoletti C	et al.	Bone carriers in diabetic foot osteomyelitis	2021	Book Chapter
Nilsson M	et al.	The composite of hydroxyapatite and calcium sulphate: A review of preclinical evaluation and clinical applications	2013	Review
Ninarello D	et al.	A comprehensive systematic review of marketed bone grafts for load-bearing critical-sized bone defects	2024	Review
Nishisho T	et al.	Locally administered zoledronic acid therapy for giant cell tumor of bone	2011	Conference Abstract
No Y	et al.	Novel injectable strontium-hardystonite phosphate cement for cancellous bone filling applications	2019	Missing one o more keywords
Nsubuga M	et al.	Surgical approach and follow-up of the complicated diabetic foot: general considerations	2024	Book Chapter
Nsubuga M	et al.	The surgical approach and follow-up of the complicated Charcot foot: general considerations	2022	Book Chapter
Oezel L	et al.	Effect of antibiotic infused calcium sulfate/ hydroxyapatite (CAS/HA) insets on implant-associated osteitis in a femur fracture model in mice	2019	No Human
Oliveira M	et al.	Ex vivo model for percutaneous vertebroplasty	2015	No Human
Oliveira M	et al.	Percutaneous vertebroplasty: a new animal model	2016	No Human
Oliver R	et al.	Comparative osteoconductivity of bone void fillers with antibiotics in a critical size bone defect model	2020	Missing one o more keywords
Oliver R	et al.	Evaluation of comparative soft tissue response to bone void fillers with antibiotics in a rabbit intramuscular model	2019	No Human
Palo N	et al.	Role of STIMULAN in chronic osteomyelitis-A randomised blinded study on 95 patients comparing 3 antibiotic compositions	2024	Missing one o more keywords
Panagopoulos P	et al.	Local antibiotic delivery systems in diabetic foot osteomyelitis: Time for one step beyond?	2015	Review
Pankratz C	et al.	Augmentation techniques for the treatment of osteoporosis-associated fractures of the extremities	2024	Missing one o more keywords

Patel H	et al.	Subchondral insufficiency fracture of the femoral head treated with core decompression and bone void filler support	2016	Missing one o more keywords
Peng S	et al.	A Bioactive Degradable Composite Bone Cement Based on Calcium Sulfate and Magnesium Polyphosphate	2024	Missing one o more keywords
Pesch S	et al.	Treatment of fracture-related infection of the lower extremity with antibiotic-eluting ceramic bone substitutes: case series of 35 patients and literature review	2020	Review
Phyo N	et al.	Medium-term outcomes of multi-disciplinary surgical management of non-ischemic diabetic heel ulcers	2021	Missing one o more keywords
Piccirilli E	et al.	Augmentation in fragility fractures, bone of contention: a systematic review	2022	Missing one o more keywords
Pierre-Jerome C	et al.	Biomechanical behavior of bone. Fractures in Charcot neuroarthropathy	2022	Book Chapter
Pignatti G	et al.	Reconstruction of severe bone defects in acetabular cup revision by application of an innovative osteoinductive paste	2013	Conference Abstract
Pilgrim M	et al.	Septic non-union of the tibial pilon: Solving an intractable problem	2024	Missing one o more keywords
Pinto A	et al.	Traumatic fractures in adults: Missed diagnosis on plain radiographs in the Emergency Department	2018	Missing one o more keywords
Pitera T	et al.	Assessment of physical performance and early treatment outcomes after implantation of modular prostheses of femoral and tibial shaft	2019	Missing one o more keywords
Pizzoli A	et al.	Efficacy of Cal-Cemex as bone substitute for tibial plateau fractures	2023	Missing one o more keywords
Poeta N	et al.	Orthobiologics for Fracture Healing in the Athlete	2021	Book Chapter
Polat B	et al.	Factors influencing the functional outcomes of tibia plateau fractures after surgical fixation	2019	Missing one o more keywords
Pountos I	et al.	Articular impaction injuries in the lower limb	2017	Missing one o more keywords
Poynter T	et al.	Intramedullary use of an antibiotic synthetic bone filler in diabetics undergoing metatarsal amputation(s)	2025	Missing one o more keywords
Prieto E	et al.	Injectable foams for regenerative medicine	2014	Missing one o more keywords
Qin C	et al.	Management of Osteomyelitis-Induced Massive Tibial Bone Defect by Monolateral External Fixator Combined with Antibiotics-Impregnated Calcium Sulphate: A Retrospective Study	2018	Missing one o more keywords
Raina D	et al.	Guided tissue engineering for healing of cancellous and cortical bone using a combination of biomaterial based scaffolding and local bone active molecule delivery	2019	Missing one o more keywords
Rajendran M	et al.	Antibiotic loaded nano rod bone cement for the treatment of osteomyelitis	2020	Missing one o more keywords
Ramanujam C	et al.	An Overview of Bone Grafting Techniques for the Diabetic Charcot Foot and Ankle	2012	Review
Reginelli A	et al.	Imaging of pediatric foot disorders	2018	Review
Ren X	et al.	Effect of structural support size and position on depressed tibial plateau fractures: A finite element analysis	2024	Missing one o more keywords

Richard R	et al.	Use of 3D Printing Technology in Fracture Management: A Review and Case Series	2023	Missing one o more keywords
Rodrigues M	et al.	Bioinspired materials and tissue engineering approaches applied to the regeneration of musculoskeletal tissues	2019	Book Chapter
Rodriguez A	et al.	Proximal Tibial Opening Wedge Osteotomy for the Treatment of Posterior Knee Instability and Genu Recurvatum Secondary to Increased Anterior Tibial Slope	2021	Missing one o more keywords
Roller B	et al.	Elution properties of a resorbable magnesium phosphate cement	2020	Missing one o more keywords
Rollo G	et al.	Bone grafiting combined with SauvÃ©-Kapandji procedures for the treatment of aseptic distal radius non-union	2020	Missing one o more keywords
Rolvien T	et al.	ß-TCP bone substitutes in tibial plateau depression fractures	2017	Missing one o more keywords
Romagnoli M	et al.	Tricalcium Phosphate as a Bone Substitute to Treat Massive Acetabular Bone Defects in Hip Revision Surgery: A Systematic Review and Initial Clinical Experience with 11 Cases	2023	Review
Rossi G	et al.	How effective is embolization with N-2-butyl-cyanoacrylate for aneurysmal bone cysts?	2017	Missing one o more keywords
Rotter R	et al.	[Operationstechnik und deren Ergebnisse der Pilon-tibiale-Fraktur]"	2017	Review
Roukis T	et al.	Joint-Sparing Surgical Management of Sanders IV Displaced Intra-Articular Calcaneal Fractures	2019	Review
Rudenko A	et al.	Local transport of antibiotics in the treatment of tubular bones chronic osteomyelitis: Literary review	2022	Review
Rupp M	et al.	Can necrotic bone be objectively identified in chronic fracture related infections? — First clinical experience with an intraoperative fluorescence imaging technique	2020	Missing one o more keywords
Ryskova L	et al.	Antibiotic-loaded Bone Cement in the Management of Neosternal Osteomyelitis	2022	Missing one o more keywords
Sahu K	et al.	Multi response optimization for compressive strength, porosity and dimensional accuracy of binder jetting 3D printed ceramic bone scaffolds	2022	Missing one o more keywords
Schlickewei C	et al.	Eluting antibiotic bone graft substitutes for the treatment of osteomyelitis in long bones. A review: Evidence for their use?	2014	Missing one o more keywords
Schmidt-Rohlfing B	et al.	Kyphoplasty and vertebroplasty : Indications, techniques, complications and results	2011	Missing one o more keywords
Schröter L	et al.	Biological and mechanical performance and degradation characteristics of calcium phosphate cements in large animals and humans	2020	Review
Sebastian S	et al.	Systemic rifampicin shows accretion to locally implanted hydroxyapatite particles in a rat abdominal muscle pouch model	2023	Missing one o more keywords
Semeshchenko D	et al.	Unconventional Therapies in Periprosthetic Joint Infections: Prevention and Treatment: A Narrative Review	2025	Review
Serrier H	et al.	Cost-effectiveness of a bone substitute delivering gentamicin in the treatment of chronic osteomyelitis of long bones: Protocol for the CONVICTION randomized multicenter study	2023	Irrelevant
Sezgin E	et al.	Biomaterials a nd Their Carriers for Managing Bone Disorders	2024	Book Chapter
Shao H	et al.	Preparation of Porous TCP Scaffolds for Cancellous Bone Engineering by 3D Gel-Printing	2021	Missing one o more keywords

Sheehy E	et al.	Antibiotic-eluting scaffolds with responsive dual-release kinetics facilitate bone healing and eliminate S. aureus infection	2025	Missing one o more keywords
Shen L	et al.	Chronic osteomyelitis treatment: A clinical and pharmaco-kinetic study of vancomycin impregnated calcium sulphate	2015	Missing one o more keywords
Shi Y	et al.	A bioactive magnesium phosphate cement incorporating chondroitin sulfate for bone regeneration	2021	Missing one o more keywords
Shibuya I	et al.	Surgical treatment of a lumbar aneurysmal bone cyst using percutaneous endoscopic lumbar discectomy	2018	Missing one o more keywords
Shiels S	et al.	Topical rifampin powder for orthopaedic trauma part II: Topical rifampin allows for spontaneous bone healing in sterile and contaminated wounds	2018	Missing one o more keywords
Siddiqui MM	et al.	Pathological Distal Tibial and Fibular Fracture in a Paediatric Patient: A Case Report	2022	Missing one o more keywords
Siemund R	et al.	Initial clinical experience with a new biointegrative cement for vertebroplasty in osteoporotic vertebral fractures	2009	Missing one o more keywords
Singh R	et al	Evaluation of Hydroxyapatite Granules Cerament and Platelet-rich Fibrin in the Management of Endodontic Apical Surgery	2020	Missing one o more keywords
Sivakumar B	et al.	Injection of a Bone Substitute in the Treatment of Unicameral Bone Cysts	2023	Missing one o more keywords
Smith T	et al.	Surgical fixation methods for tibial plateau fractures	2024	Missing one o more keywords
Stanley C	et al.	Salvage of a Comminuted Proximal Tibial Polymicrobial Infected Non-Union with Antibiotic Loaded Bio-Composite and Intramedullary Nailing: A Case Report	2024	Missing one o more keywords
Staruch R	et al.	Injectable Pore-Forming Hydrogel Scaffolds for Complex Wound Tissue Engineering: Designing and Controlling Their Porosity and Mechanical Properties	2017	Missing one o more keywords
Stone T	et al.	Letter to the Editor: Percutaneous Doxycycline Treatment of Aneurysmal Bone Cysts With Low Recurrence Rate: A Preliminary Report	2023	Letter
Stravinskas M	et al.	Vancomycin elution from a biphasic ceramic bone substitute	2019	Missing one o more keywords
Stravinskas M	et al.	A ceramic bone substitute containing gentamicin gives good outcome in trochanteric hip fractures treated with dynamic hip screw and in revision of total hip arthroplasty: A case series	2018	Missing one o more keywords
Stravinskas M	et al.	Pharmacokinetics of gentamicin eluted from a regenerating bone graft substitute in vitro aNd cliNical ReleaSe StudieS	2016	Missing one o more keywords
Stravinskas M	et al.	Antibiotic Containing Bone Substitute in Major Hip Surgery: A Long Term Gentamicin Elution Study	2018	Missing one o more keywords
Sun M	et al.	Biodegradable intramedullary nail (BIN) with high-strength bioceramics for bone fracture	2021	Missing one o more keywords
Suresh I	et al.	A COMPARATIVE ANALYSIS OF ELEVATION OF DEPRESSED TIBIAL CONDYLE FRACTURE BY AUTOGENOUS BONE GRAFT AND HYDROXY APATITE CRYSTALS	2022	Missing one o more keywords

Sykes M	et al.	Infected open calcaneal fractures – A delayed reconstruction technique using calcium sulphate and hydroxyapatite antibiotic elucidating void filler	2020	Missing one o more keywords
Tadje J	et al.	Embolism After Injection of a Calcium Sulfate Bone Graft Substitute: A Case Report	2022	Missing one o more keywords
Tanodekaew S	et al.	Physico-chemical properties and biocompatibility of in situ-hardening polylactide/nano hydroxyapatite composite for bone substitute	2023	Missing one o more keywords
Tanwar Y	et al.	The role of bioactive glass in the management of chronic osteomyelitis: a systematic review of literature and current evidence	2020	Review
Tao Z	et al.	Combined treatment with parathyroid hormone (1â€"34) and beta-tricalcium phosphate had an additive effect on local bone formation in a rat defect model	2016	Missing one o more keywords
Tao Z	et al.	Treatment study of distal femur for parathyroid hormone (1-34) and Î²-tricalcium phosphate on bone formation in critical-sized defects in osteopenic rats	2015	Missing one o more keywords
Tao Z	et al.	Effect exerted by Teriparatide upon Repair Function of Î²-tricalcium phosphate to ovariectomised rat's femoral metaphysis defect caused by osteoporosis	2015	Missing one o more keywords
Tarantino U	et al.	Surgical therapy: Vertebro-cifoplastic: Pros and cons	2018	Book Chapter
Tatara A	et al.	Tissue engineering in orthopaedics	2016	Missing one o more keywords
Telis A	et al.	Treatment of tibial plateau fractures with a novel fenestrated screw system for delivery of bone graft substitute	2021	Missing one o more keywords
Teotia A	et al.	Endogenous Platelet-Rich Plasma Supplements/Augments Growth Factors Delivered via Porous Collagen-Nanohydroxyapatite Bone Substitute for Enhanced Bone Formation	2019	Missing one o more keywords
Terzi S	et al.	Efficacy and Safety of Selective Arterial Embolization in the Treatment of Aneurysmal Bone Cyst of the Mobile Spine	2017	Missing one o more keywords
Tian X	et al.	Co-delivery of rhBMP-2 and zoledronic acid using calcium sulfate/hydroxyapatite carrier as a bioactive bone substitute to enhance and accelerate spinal fusion"	2024	Missing one o more keywords
Tian X	et al.	Evaluation of an Injectable Biphasic Calcium Sulfate/Hydroxyapatite Cement for the Augmentation of Fenestrated Pedicle Screws in Osteoporotic Vertebrae: A Biomechanical Cadaver Stud	2022	Missing one o more keywords
Tiruveedhula M	et al.	A 2-Stage Approach in Managing Diabetic Forefoot Ulcers	2022	Missing one o more keywords
Toepfer A	et al.	Allogenic Cancellous Bone versus Injectable Bone Substitute for Endoscopic Treatment of Simple Bone Cyst and Intraosseous Lipoma of the Calcaneus and Is Intraosseous Lipoma a Developmental Stage of a Simple Bone Cyst?	2023	Missing one o more keywords
Tomasian A	et al.	Benign Bone Tumors: State of the Art in Minimally Invasive Percutaneous Interventions"	2023	Review
Tonnelet R	et al.	Aneurysmal bone cyst of thoracic vertebrae in a young asymptomatic boy with spinal cord compression. Successful treatment by percutaneous approach with PMMA-cement	2019	Missing one o more keywords
Truedsson A	et al.	Osseointegration of Titanium Implants in Onlay of Cerament™, a New Ceramic Bone Substitute	2016	No Human
Truedsson A	et al.	Bone substitute as an on-lay graft on rat tibia	2010	No Human
Tsoumakidou G	et al.	Magnetic Resonance Imaging in degenerative disease of the lumbar spine: Fat Saturation technique and contrast medium	2018	Missing one o more keywords

Tsukamoto S	et al.	Imaging of Spinal Bone Tumors: Principles and Practice	2022	Missing one o more keywords
Turzańska K	et al.	Improving the Management and Treatment of Diabetic Foot Infection: Challenges and Research Opportunities	2023	Review
Unsworth A	et al.	Local Antimicrobial Therapy with Combined Aminoglycoside and Vancomycin Compared to Aminoglycoside Monotherapy in the Surgical Management of Osteomyelitis and Fracture-Related Infection	2024	Missing one o more keywords
Vanni D	et al.	Alternatives to PMMA for vertebral augmentation procedures	2020	Book Chapter
Vasavada K	et al.	The Effect of Bone-Void Filler on Anterior Knee Pain Following BPTB Autograft ACL Reconstruction	2022	Conference Abstract
Vegt P	et al.	The photodynamic bone stabilization system: A minimally invasive, percutaneous intramedullary polymeric osteosynthesis for simple and complex long bone fractures	2014	Missing one o more keywords
Venkateswaran V	et al.	Antibiotic Eluting Bone Void Filler Versus Systemic Antibiotics For Pedal Osteomyelitis	2025	Missing one o more keywords
Vicente M	et al.	Septic Nonunion Caused by Mycobacterium canariasense	2018	Missing one o more keywords
Völk D	et al.	Outcome after polyaxial locking plate osteosynthesis in proximal tibia fractures: a prospective clinical trial	2021	Missing one o more keywords
Von Rüden C	et al.	Failure of fracture fixation in osteoporotic bone	2016	Missing one o more keywords
Wang G	et al.	Custom-Made Antibiotic Cement-Coated Nail for the Treatment of Infected Bone Defect	2021	Missing one o more keywords
Wang J	et al.	Local Antibiotic Delivery Systems: Current and Future Applications for Diabetic Foot Infections	2018	Missing one o more keywords
Wang L	et al.	Efficacy comparison of antibiotic bone cement–coated implants and external fixations for treating infected bone defects	2023	Missing one o more keywords
Wang M	et al.	Effect of an α-calcium sulfate hemihydrate/treated dentin matrix composite to regenerate bone in critically sized SD rat calvarial defects	2024	Missing one o more keywords
Wang R	et al.	Comparison of the Clinical Outcomes of Vesselplasty vs. Percutaneous Vertebroplasty for the Treatment of Neurologically Intact Osteoporotic KÃ¼mmellâ€™s Disease: A Retrospective Study	2024	Missing one o more keywords
Wang Y	et al.	Management of the Cavity After Removal of Giant Cell Tumor of the Bone	2021	Review
Wang Z	et al.	Structural Bicortical Autologous Iliac Crest Bone Graft Combined with the Tunnel Bone Tamping Method for the Depressed Tibial Plateau Fractures	2021	Missing one o more keywords
Wangqin R	et al.	Complete resolution of a cervical spine aneurysmal bone cyst after single session of endovascular embolization: Case report	2019	Missing one o more keywords
Wassif R	et al.	Recent advances in the local antibiotics delivery systems for management of osteomyelitis	2021	Missing one o more keywords
Wee J	et al.	The role of orthobiologics in foot and ankle surgery: Allogenic bone grafts and bone graft substitutes	2017	Review
Whisstock C	et al.	Multidisciplinary approach for the management and treatment of diabetic foot infections with a resorbable, gentamicin-loaded bone graft substitute	2020	Missing one o more keywords

Wu C	et al.	Limitation of the antibiotic-eluting bone graft substitute: An example of gentamycin-impregnated calcium sulfate	2018	Missing one o more keywords
Wu C	et al.	Injectable and biodegradable composite bone filler composed of poly(propylene fumarate) and calcium phosphate ceramic for vertebral augmentation procedure: An in vivo porcine study	2017	Missing one o more keywords
Wu J	et al.	Biocompatibility of sextant minimally invasive pedicle screw fixation for osteoporotic vertebral fractures in the elderly	2016	Missing one o more keywords
Wu Z	et al.	Comparison of cement leakage rate and severity after percutaneous vertebroplasty for osteoporotic vertebral compression fractures using front-opening versus side-opening cannulas	2021	Missing one o more keywords
Xue N	et al.	Bone Tissue Engineering in the Treatment of Bone Defects	2022	Review
Yan F	et al.	Biphasic Injectable Bone Cement with Fe(3)O(4)/GO Nanocomposites for the Minimally Invasive Treatment of Tumor-Induced Bone Destruction	2019	Missing one o more keywords
Yang G	et al.	Progress of calcium sulfate and inorganic composites for bone defect repair	2013	Review
Yang H	et al.	Bone healing response to a synthetic calcium sulfate/β-tricalcium phosphate graft material in a sheep vertebral body defect model	2012	Missing one o more keywords
Yang Y	et al.	Efficacy of robot-assisted core decompression combined with human umbilical cord-derived mesenchymal stem cell transplantation for osteonecrosis of the femoral head	2022	Missing one o more keywords
Yeo Q	et al.	Use of a biphasic cement bone substitute in the management of metaphyseal fractures	2019	Missing one o more keywords
Young B	et al.	Microbial Persistence, Replacement and Local Antimicrobial Therapy in Recurrent Bone and Joint Infection	2023	Missing one o more keywords
Yousefi A	et al.	A review of calcium phosphate cements and acrylic bone cements as injectable materials for bone repair and implant fixation	2019	Review
Yu A	et al.	Outcomes of distal femoral replacement versus internal fixation for metastatic bone diseases of the distal femur	2024	Missing one o more keywords
Yu S	et al.	Kirschner Wire and Bone Cement is a Viable Alternative to Reconstruction of Large Iliac Bone Defects after Strut Bone Graft Harvesting	2017	Missing one o more keywords
Zampelis V	et al.	The effect of a biphasic injectable bone substitute on the interface strength in a rabbit knee prosthesis model.	2013	Missing one o more keywords
Zapata D	et al.	Nanotechnology in the Diagnosis and Treatment of Osteomyeliti	2022	Review
Zarzour O	et al.	CT-guided radiofrequency ablation in patients with aneurysmal bone cysts	2018	Missing one o more keywords
Zhan W	et al.	Use of a raft construct through a locking plate without bone grafting for split-depression tibial plateau fractures	2015	Missing one o more keywords
Zhong C	et al.	Advances in the antimicrobial treatment of osteomyelitis	2023	Review
Zhou H	et al.	Monetite an important calcium phosphate compoundâ€"Its synthesis properties and applications in orthopedics	2021	Review
Zhou J	et al.	Moringa Oleifera-Incorporated Nanofibrous Composite Bone Filler Promotes Critical Sized Bone Defect Healing in a Rat Model: An In Vitro and In Vivo Study	2023	Missing one o more keywords
Zhou J	et al.	Using Ni-Ti shape memory alloy vertebral reduction fixator to treat vertebral compression fractures	2012	Missing one o more keywords
Zhou L	et al.	Innovative Tissue-Engineered Strategies for Osteochondral Defect Repair and Regeneration: Current Progress and Challenges	2020	Review

Zhou Y	et al.	Comparison of Therapeutic Effects of PVP and PKP Combined With Triple Medication on Mild and Moderate Osteoporotic Vertebral Compression Fracture in the Elderly	2022	Missing one o more keywords
Zhu T	et al.	Novel bioactive glass based injectable bone cement with improved osteoinductivity and its in vivo evaluation	2017	Missing one o more keywords
Ziroglu N	et al.	Modified and alternative bone cements can improve the induced membrane: Critical size bone defect model in rat femur	2024	Missing one o more keywords
Zivanovic D	et al.	Elastic stable intramedullary nailing of humerus fractures in children	2018	Review
Zrodowski M	et al.	The use of bone substitute (CERAMENT) in the treatment of benign bone tumors and tumor-like lesions	2022	No Open Access
	et al.	CERAMENT™	Bone Void Filler Device Registry	2020	No Open Access
